# Leveraging machine learning to analyze sentiment from COVID‐19 tweets: A global perspective

**DOI:** 10.1002/eng2.12572

**Published:** 2022-09-18

**Authors:** Md Mahbubar Rahman, Nafiz Imtiaz Khan, Iqbal H. Sarker, Mohiuddin Ahmed, Muhammad Nazrul Islam

**Affiliations:** ^1^ Department of Computer Science and Engineering Military Institute of Science and Technology (MIST) Dhaka Bangladesh; ^2^ Department of Computer Science and Engineering Chittagong University of Engineering and Technology Chittagong Bangladesh; ^3^ School of Science Edith Cowan University Joondalup Western Australia Australia

**Keywords:** coronavirus, COVID‐19, deep neural network, machine learning, outbreak, pandemic, prediction, sentiment analysis, social media

## Abstract

Since the advent of the worldwide COVID‐19 pandemic, analyzing public sentiment has become one of the major concerns for policy and decision‐makers. While the priority is to curb the spread of the virus, mass population (user) sentiment analysis is equally important. Though sentiment analysis using different state‐of‐the‐art technologies has been focused on during the COVID‐19 pandemic, the reasons behind the variations in public sentiment are yet to be explored. Moreover, how user sentiment varies due to the COVID‐19 pandemic from a cross‐country perspective has been less focused on. Therefore, the objectives of this study are: to identify the most effective machine learning (ML) technique for classifying public sentiments, to analyze the variations of public sentiment across the globe, and to find the critical contributing factors to sentiment variations. To attain the objectives, 12,000 tweets, 3000 each from the USA, UK, and Bangladesh, were rigorously annotated by three independent reviewers. Based on the labeled tweets, four different boosting ML models, namely, CatBoost, gradient boost, AdaBoost, and XGBoost, are investigated. Next, the top performed ML model predicted sentiment of 300,000 data (100,000 from each country). The public perceptions have been analyzed based on the labeled data. As an outcome, the CatBoost model showed the highest (85.8%) F1‐score, followed by gradient boost (84.3%), AdaBoost (78.9%), and XGBoost (83.1%). Second, it was revealed that during the time of the COVID‐19 pandemic, the sentiments of the people of the three countries mainly were negative, followed by positive and neutral. Finally, this study identified a few critical concerns that impact primarily varying public sentiment around the globe: lockdown, quarantine, hospital, mask, vaccine, and the like.

## Graphical abstract

Variation of public sentiment over time for Bangladeshi people.

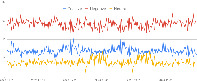

## 1 INTRODUCTION

COVID‐19 is an infectious disease identified in Wuhan, China, in December 2019.^1^ Soon after the identification, the virus spread worldwide, affecting millions of people's lives. The World Health Organization (WHO)* has labeled this COVID‐19 as a public health emergency of international concern on January 30, 2020 and a pandemic on March 11, 2020.^2^ From the beginning to now, all the countries are pushing their limits to curb the spread as much as possible. Apart from creating a pandemic, this virus also created an unprecedented socio‐technological crisis in our society. Although the world has witnessed pandemics before, all of these pandemics were in the pre‐social media era.^3^, ^4^ Thus, the researchers had little chance to analyze public sentiment during past pandemics. On the other hand, in the current era of social media, millions of people worldwide have the opportunity to use social media platforms to express their views, opinions, and feelings.^5^ As social distancing is the only way to prevent the spread of COVID‐19, social media platforms like Facebook and Twitter, have become the sole option for people to express their sentiments. Thus, the authorities and law enforcement agencies are taking direct inputs from the social media trends and implementing appropriate measures like quarantine, isolation, and so forth, to fight against the spread of the virus. Hence, understanding and analyzing the public sentiment due to the COVID‐19 pandemic becomes a key concern for the researchers to explore and extract different views of many people related to education, business, the environment, and various social issues.

The COVID‐19 pandemic creates countrywide partial or complete lockdown in many countries around the world. To curb the spreading of the coronavirus, the government of many countries has advised the mass people to maintain social distancing, home quarantine, wear masks, and so forth. As a result, social media has become extremely important for sharing real‐time information, advice, feelings, emotions, and opinions from experts and people around the world. The exchange of huge data related to COVID‐19 in social media created the concerns of researchers, and some studies are carried out focusing the sentiment analysis using Twitter data.^6^, ^7^ Few have conducted sentiment analyses during the COVID‐19 pandemic; for example, Islam et al.^8^ studied the public perceptions toward COVID‐19 and Black Fungus, Chandra and Krishna^9^ analyzed public sentiment regarding COVID‐19 by deep learning methods. However, the reasons behind the change in public perceptions during the COVID‐19 pandemic are yet to be explored. Again, most of the studies analyzed the sentiment from COVID‐19 tweets for a specific country context,^7^, ^10^ and a very few have paid attention to explore the public sentiment from a cross‐country perspective.^11^ Moreover, different studies adopted different machine learning (ML) techniques to analyze the public sentiment,^12^, ^13^, ^14^, ^15^ a few focused on adopting multiple ML techniques in a single study to find out the best performed one and analyzed the tweets using the best‐performed technique.^16^ Apart from these, since most of the studies were published in the early or middle stage to analyze public sentiment, thus these studies did not capture the outline of the COVID‐19 pandemic.

Therefore, the objectives of this research are: first, to explore the ML techniques for finding out the best‐performed technique for COVID‐19‐related tweet sentiment analysis. Second, to analyze and investigate user sentiments across the countries (UK, USA, and Bangladesh) due to the COVID‐19 pandemic. Third, to explore and highlight some critical concerns that primarily impact the sentiments across three countries due to the COVID‐19 pandemic. To attain these objectives, the authors have explored different ML techniques to analyze public sentiment based on the data collected from January to December 2020. The study also focused on finding the best ML models based on the performance and the sentiment variation in three different countries and the possible reasons behind the change in public sentiment during the COVID‐19 pandemic from a cross‐country perspective.

The rest of this article is organized as follows. Section 2 covers the overview of past studies related to sentiment analysis. The methodology to conduct data analysis is discussed in Section 3, while Section 4 presents the overall development and implementation of different ML models. Lastly, Section 5 provides the data analysis and visualization in broader heads. Section 6 concludes the article with discussions and limitations.

## 2 LITERATURE REVIEW

This section briefly discusses the studies carried out during the COVID‐19 pandemic on sentiment analysis. For example, Abd‐Alrazaq et al.^17^ identified the main topics posted on Twitter related to the COVID‐19 pandemic by using latent Dirichlet allocation (LDA). In a similar study, Qazi et al.,^18^ the influence of information sources on situational awareness for public health is evaluated during the COVID‐19 pandemic to adopt health‐protective behaviors such as social distancing.

Sentiment analysis of COVID‐19‐related Twitter data is also performed from a cross‐country perspective. For example, in Reference 19, the authors studied tweets from 12 different countries from different geographical locations. Here, the authors analyzed the Twitter sentiments and emotions using NRC Emotion Lexicon^20^ to understand how the citizens of different countries are dealing with the pandemic situation. The impacts of the COVID‐19 pandemic were not confined to the health sector, in addition, the economic sector suffers different new challenges due to the rapid spread of coronavirus all over the world.

A few studies were carried out to investigate the economic risk in stock markets, oil price volatility, geopolitical risk, and economic policy uncertainty during the COVID‐19 pandemic.^4^, ^21^, ^22^ Rajput et al.^6^ presented a statistical analysis of the Twitter messages related to COVID‐19 posted since January 2020. The study is conducted based on word frequencies and sentiments of the individual tweet messages. The study found the majority of tweets as positive (90%) polarity compared with negative (15%) polarity. The another study^23^ was conducted in the Philippines to reveal the sentiment of the Filipinos due to extreme community quarantine. This research uses a qualitative approach to analyze the effects of the pandemic on a personal lifestyle based on the tweets of the users in a particular area.

Again, in Reference 7, tweets from 12 different countries are analyzed for sentiment and emotions analysis aiming to understand how the citizens of different countries are dealing with the pandemic situation. The study found that countries like Belgium (63%), India (60%), and Australia were tweeting about COVID‐19 with a positive sentiment whereas people in China had negative (55%) sentiment. It can be found that a few studies^11^, ^19^ have been conducted from the cross country perspective from the different geological locations. Nonetheless, there are studies that were carried out focusing on different context such as place,^24^ academia,^25^ particular effects^22^, ^26^ due to this COVID‐19 pandemic. The wildfire of COVID‐19 has brought the researchers to uncover many hidden themes in tweet data that represent the pandemic's most pressing issues.^27^ The mass people are taking input regarding new concerns from different formal and informal information sources to tackle this pandemic situation in the public health sector. A summary of the few other related articles is presented in Table 1.

**TABLE 1:** Summary of the few example articles conducted for sentiment analysis

Ref.	Objectives	Technology used	Data description	Context
29	To measure disease activity through sentiment analysis for H1N1 swine flu pandemic	Support vector regression	Two twitter datasets are used. First dataset contains 951,697 tweets, second one contains 4,199,166 tweets	Study conducted in USA
30	To classify Chikungunya and Dengue related Twitter data	Naive Bayes classifier	4037 tweets data	Study conducted in India
31	To analyze sentiment toward the safety of the H1N1 vaccine	Online survey	Text opinions of 27,382 participants	Study conducted in Canada
32	To predict epidemic‐prone areas through sentiment analysis	SVM, naive Bayes, RNN‐LSTM	2888 tweets data	
33	To analyze sentiment for monitoring disease and vaccination against H1N1 pandemic influenza	Computer assisted telephone interview	13,010 participants aged 14 or higher	Study conducted in Germany
34	To analyze public perceptions on vaccine and antiviral uptake	Protection motivation theory (PMT)	14,312 tweets data	Study conducted in UK
35	To perform lexicon‐based sentiment analysis for classifying both tagged and untagged twitter	Lexicon based approach, SVM	93,447 tweets data	

The related studies show that sentiment analysis during this COVID‐19 pandemic is a crucial and important issue to analyze and investigate. However, the pandemic has a contextual impact because the pandemic does not spread consistently in various nations and the population density, age group, and emotional ability of the population differ from country to country. Again, sufficient data are required to get accurate and reliable findings from the study. Apart from the COVID‐19 pandemic, sentiment analysis research on previous pandemics/epidemics and other issues have also been conducted.

In summary, the following key concerns have been identified. First, most research has focused on social media data to analyze public sentiment. Second, for sentiment analysis primarily positive, negative, and neutral sentiment, and in a few cases, different emotions like joy, sad, fear, anger, and so forth, were measured. Third, a number of studies were conducted related to sentiment analysis from Twitter data, their outcomes are always varied from place to place, and location to location.^6^, ^26^ Fourth, some studies^24^, ^25^, ^26^ are conducted from a particular country perspective. Fifth, no study has been found to predict user sentiment during the time of COVID‐19 by boosting ensemble methods, which refers to a collection of algorithms that help slow learners become strong.^28^ Finally, only a few studies have been conducted to analyze public sentiment from a cross‐country/global perspective. Thus, to fulfill these research gaps, different boosting algorithms were explored for analyzing the tweet data while analyzing the sentiments from COVID‐19 tweets from a global perspective.

## 3 RESEARCH METHODOLOGY

This section briefly discusses the steps that have been followed in this study. The research methodology includes the following:

- *Data acquisition*: The study contains only English language tweets. The coronavirus‐related tweets data was collected from Twitter using a publicly available application programming interface (API) from three different countries, namely, the UK, the USA, and Bangladesh, due to significant infection rates as well as different geographical locations from Europe, North America, and Asia. The Twitter standard search API used a set of predefined search terms which are the most widely used scientific and news media terms relating to the novel coronavirus like “COVID‐19”;, “coronavirus”;, “lockdown”;, “isolation”;, “quarantine”;, “pandemic”;, and “2020‐nCov”;. The summary of the dataset is presented in Table 2. From each country, 100,000 tweets were retrieved, while 4000 tweets from each studied country were annotated manually for training the ML models. These tweets were posted between January 1, 2020 and December 31, 2020. The extracted tweets data contains text, metadata, user handle, location, date, language, timestamp, and user profile information, including the number of followers, likes, and retweets. Since the metadata of the tweets changes over time, we had to recollect the tweets using the old tweet ID at the end of the study period. Twitter standard search API allows access to old tweets using tweet IDs. The tweet ID is also stored and acts as the primary key, so that duplicate tweets are not stored.
- *Data preprocessing*: The extracted tweets were in unstructured format due to informal conversations, short messages, signs, grammatical errors, and abbreviations used by Twitter users. Thus, data preprocessing is required to eliminate all special characters and unstructured forms before feeding into the ML classifiers. The acquired data were preprocessed by converting tweets to lowercase characters and removing usernames, URLs, punctuation, links, tabs, and spaces. The stop words like “a”;, “an”;, and “the”; are removed as they carry little meaning in a sentence. Next, all the contractions in the tweets are expanded. Tweet data obtained from Twitter usually contains a lot of HTML entities that are also removed. The non‐English tweets were identified by checking the language field in the tweet's metadata and removed from the analysis.
- *Data labeling*: The annotators were given a total of 12,000 unlabeled Twitter data to prepare a training set for ML models. These annotators labeled the same set of tweets into three classes according to sentiments expressed/observed in the tweets: positive, negative, and neutral. The annotators followed the following heuristics in the labeling process:
  1. Positive: If the entire tweet has a positive/happy/excited/joyful attitude or if something is mentioned with positive connotations. Also, if more than one sentiment is expressed in the tweet but the positive sentiment is more dominant.
  2. Negative: If the entire tweet has a negative/sad/displeased attitude or if something is mentioned with negative connotations. Also, if more than one sentiment is expressed in the tweet but the negative sentiment is more dominant.
  3. Neutral: If the creator of the tweet expresses no personal sentiment/opinion in the tweet and merely transmits information. Advertisements of different products would be labeled under this category.
  **TABLE 2:** Summary of the dataset

Name of the country	Number of manually annotated tweets	Total number of tweets	Unique words in tweets	Valid words count	Max occurred word (COVID‐19) count
USA	4000	100,000	472,000	900,005	85,743
UK	4000	100,000	409,000	800,700	94,952
Bangladesh	4000	100,000	396,000	700,430	74,459
Combined	12,000	300,000	981,000	2,401,135	255,154
  Besides, the annotators were instructed to keep personal biases out of labeling and make no assumptions, that is, judge the tweet not from any past extra personal information and only from the information provided in the current individual tweet. Moreover, the annotators were working separately in their quiet time to reduce the Hawthorne effect^36^ in the annotation process. The annotation measured from three different annotators was combined through a majority vote to get an average opinion. We also calculated the human‐human agreement for labeling the tweet. Some accepted inter‐rater reliability techniques^37^, ^38^ are used to find out the inter‐annotator agreement coefficient. First, the percent of agreement is applied on inter‐annotator labels. As each tweet belongs to three labels, namely, positive, negative, and neutral, the inter‐annotator agreement was calculated for each label then an average was taken for these three labels. The pairwise agreement is also calculated for each pair of annotators. The average agreement is 85%; which shows a strong agreement among the annotators. Agreement between pairs of the three annotators is almost uniform. Again, the inter‐annotator agreement could be evaluated by Fleiss' kappa and Krippendorf's alpha.^39^ The general formula for these coefficients is as follows:
(1) $$\kappa =\frac{p_{0}-p_{e}}{1-p_{e}},$$
  where $p_{o}$ is observed agreement and $p_{e}$ expected agreement by chance if the annotators pick the labels randomly. The summary of the inter‐rater agreement coefficient values is presented in Table 3.
  **TABLE 3:** Statistics of inter‐annotator agreement coefficients of labeled dataset

Applied method	Annotators	Positive	Negative	Neutral	Average
Percentwise agreement	A1 vs. A2	0.85	0.89	0.86	0.85
	A1 vs. A3	0.92	0.91	0.89	
	A2 vs. A3	0.76	0.79	0.81	
	Average	0.84	0.86	0.85	
Krippendorf's alpha	α	0.86	0.84	0.79	0.83
Fleiss kappa	κ	0.85	0.81	0.79	0.81
	Observed	0.79	0.75	0.66	0.73
	Expected	0.31	0.59	0.56	0.48
  The average inter‐rater agreement coefficient values of Krippendorf's alpha and Fleiss kappa are 83% and 81%, respectively. It is considered that inter‐rater agreement coefficient values over 0.8 are outstanding.^39^ The coefficient values of different inter‐rater reliability techniques show that the conducted annotation process is consistent and reliable.
- *Data embedding*: A computer does not understand the semantics of the text, as well as the ML models, cannot process text data, thus it is essential to convert these textual data into numerical data. Here, sentence embedding techniques were used where the sentences are represented as vectors with their semantic meaning; because in the case of large text in a sentence word embedding would be tedious and limited to extracting necessary information. Again, several sentence embedding techniques are available in NLP research like word2Vec,^†^, BERT,^‡^ and Universal Sentence Encoder (USE).^§^ In this research, USE is adopted that encodes the text into high dimensional vectors and outperforms other pretrained word embedding models.^40^ The preprocessed tweet data, as discussed above, is converted to the numeric vector using the USE. Then, these numeric vectors are fed into different supervised ML models to determine the sentiment and classified into the different sentiment class labels named positive, negative, and neutral.
- *Data oversampling*: In the training dataset, there was a class imbalance problem as the quantity of positive, negative, and neutral tweets were not the same. The class distribution of the labeled tweets showed that the number of negative tweets is higher than positive and neutral tweets whereas the number of positive tweets is larger than neutral (see Figure 1A). An adaptive synthetic (ADASYN)^41^ sampling method was applied to have a similar number of instances for all three classes. The basic concept behind ADASYN is to use a weighted distribution for different minority class examples based on learning difficulty. This oversampling method produces more synthetic data of minority class examples that have more difficulty learning by the classifiers. After applying the oversampling method, the class distribution of the tweets is presented in Figure 1B and shows that biases in the classes were removed.
- *Training and evaluating the models*: In this phase, four different boosting algorithms are trained by performing hyperparameter tuning. After training, the models are evaluated in terms of precision, recall, and F1‐score. The model evaluation phase is articulated in the next section.
- *Analyzing public sentiment*: In this phase, the public sentiment of three different countries, BD, UK, and USA, has been analyzed with the help of the best‐performed ML model. Here, the best‐performed ML mode is used to predict the class labels of the unlabeled tweets. Next, by having the class labels of the unlabeled tweets, the overall public sentiment of the said countries has been analyzed.

**FIGURE 1:**
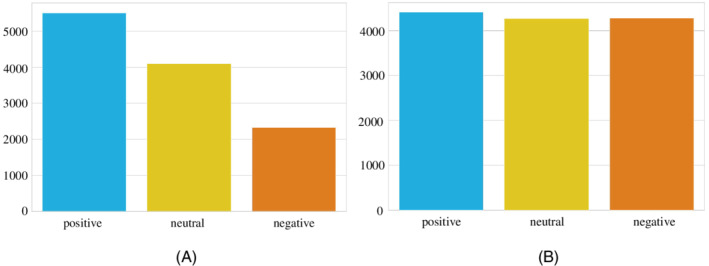
Sentiment count of all the labeled tweets: (A) Before sampling and (B) after sampling

## 4 PERFORMANCE ANALYSIS OF ML MODELS

This section briefly discusses the exploration of four different boosting algorithms for the classification of user sentiment, including CatBoost (CB), AdaBoost (AB), gradient boosting (GB), and XGBoost (XB). The primary aim of developing four ML models considering these four algorithms was to find the best predictive model for social media data sentiment classification. The procedures adopted for data collection, preprocessing, annotation, embedding, and oversampling are discussed in the previous section to develop these models.

The ML models are used for analyzing the sentiment through the tweet dataset. The analyzed and evaluated results are visualized through different kinds of graphical means like receiver operating characteristic (ROC) curve on both training and testing dataset, evaluation metrics like precision, recall, F1‐score, confusion matrix, training, and validation loss. As mentioned earlier, the ML models used pretrained language model USE to represent the tweet data as numeric vectors for training and testing purposes. A random train–test split combination of 80–20 was applied to the manually labeled dataset, as this combination is mostly used in recent studies by ML researchers for splitting the dataset.^42^, ^43^, ^44^, ^45^, ^46^, ^47^ In the 80–20 splitting combination, 80% data is considered a training dataset, while 20% data is considered a test dataset. The hyperparameters were tuned using the grid search tuning method^48^ to find out the appropriate hyperparameters for the applied models. The hyperparameters that are tuned for each of the algorithms are elucidated in Table 4. The performance of the models was analyzed based on precision, recall, and F1‐score.

**TABLE 4:** Statistics of inter‐annotator agreement coefficients of labeled dataset

Model	Hyper‐parameters	Value
AdaBoost	base	RandomForestClassifier()
	base_estimators	base
	n_estimators	100
	random_state	500
CatBoost	iterations	1000
	learning_rate	0.1
XgBoost	n_estimators	100
	random_state	700
Gradient boost	random_state	1000
	learning_rate	0.1
	n_estimators	500

The models were trained based on train data, whereas each model's performance was analyzed on both train and test sets. Nonetheless, macro averaging of the classes was used to calculate the final evaluation metric for each algorithm. As the oversampling method was used to remove the class imbalance problem, accuracy, which is a popular choice to evaluate the performance of a classifier, was not used as an evaluation metric as the prior studies^49^ showed that it is not the correct metric to use where sampling method is utilized. The developed models are briefly discussed in the following subsections.

**FIGURE 2:**
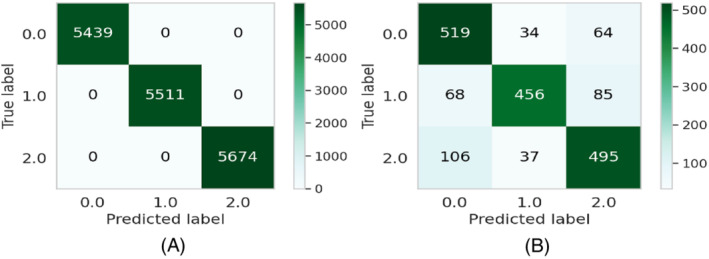
Confusion matrices for the AB model: (A) Train data and (B) test data

**FIGURE 3:**
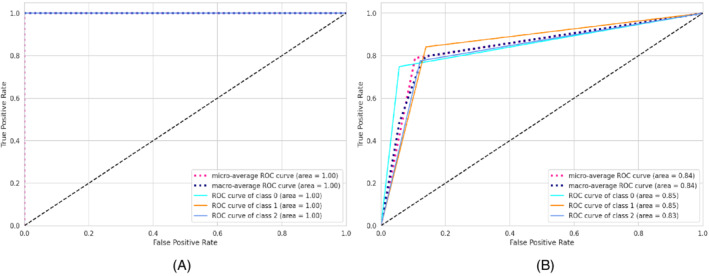
ROC curves for the AB model: (A) Train data and (B) test data

**FIGURE 4:**
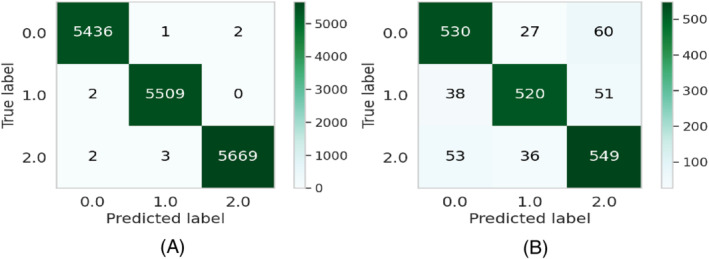
Confusion matrices for the CB model: (A) Train data and (B) test data

**FIGURE 5:**
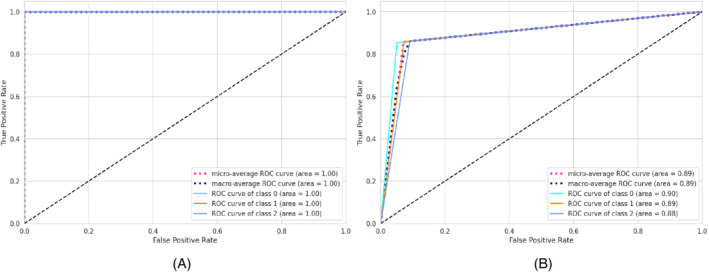
ROC curves for the CB model: (A) Train data and (B) test data

**FIGURE 6:**
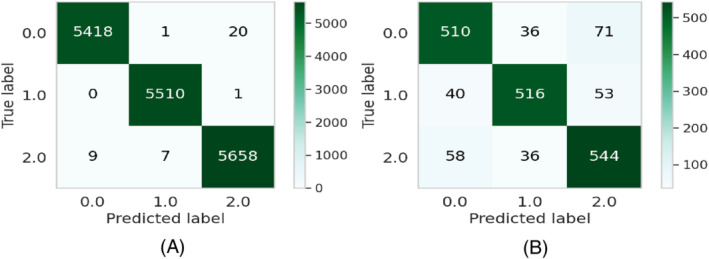
Confusion matrices for the GB model: (A) Train data and (B) test data

**FIGURE 7:**
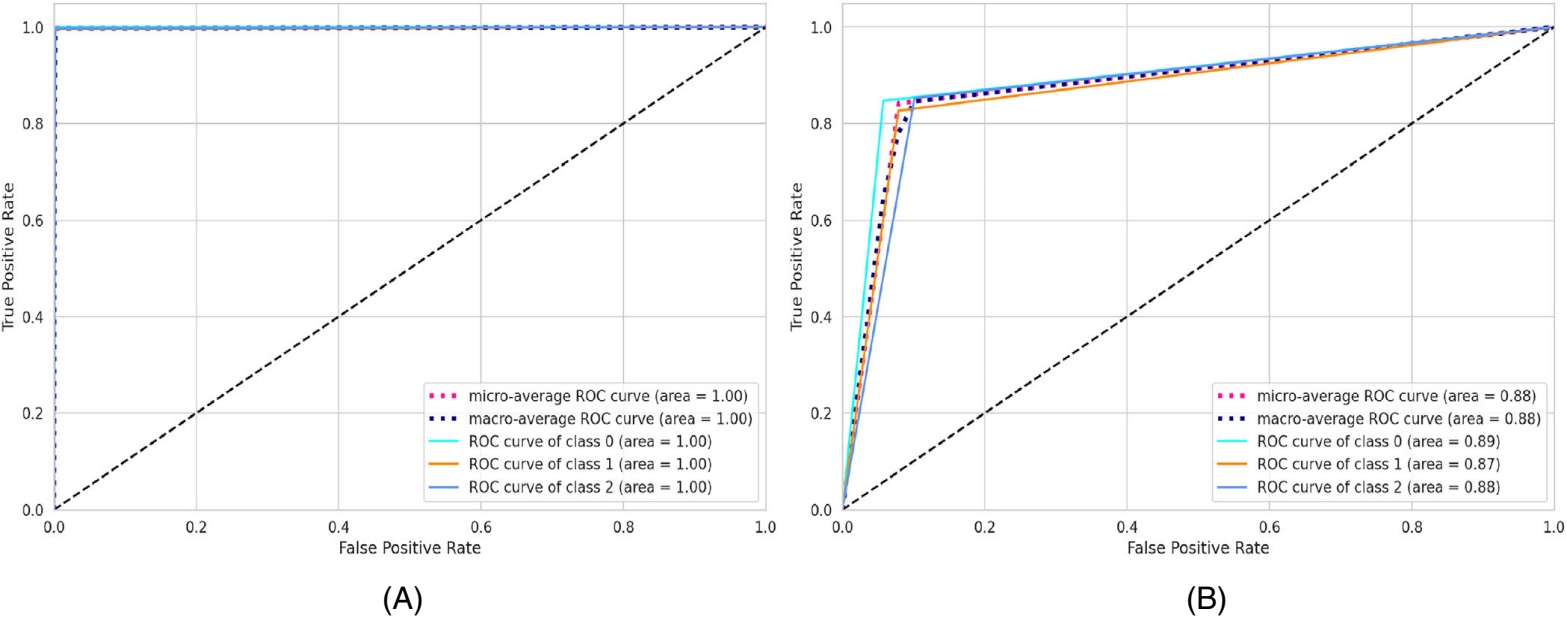
ROC curves for the GB model: (A) Train data and (B) test data

**FIGURE 8:**
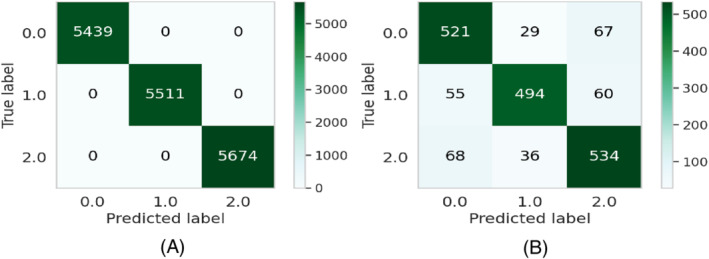
Confusion matrices for the XB model: (A) Train data and (B) test data

**FIGURE 9:**
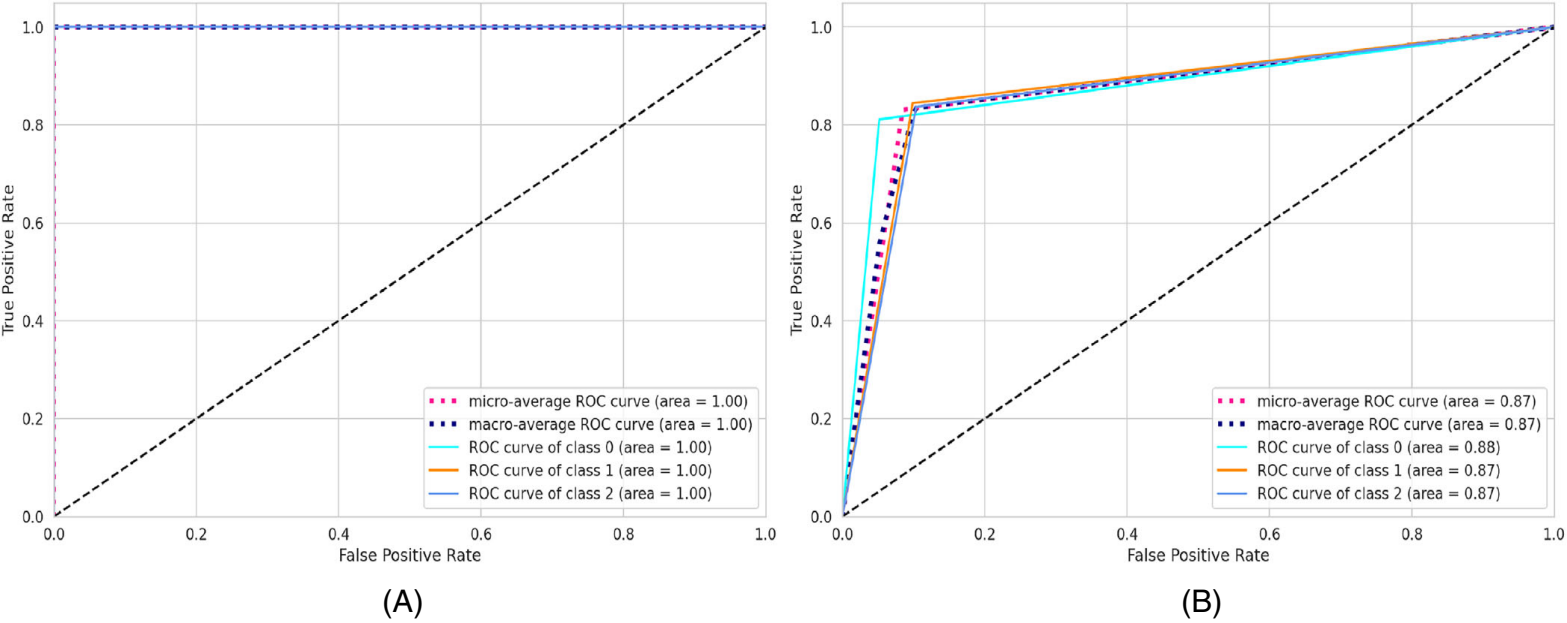
ROC curves for the XB model: (A) Train data and (B) test data

**FIGURE 10:**
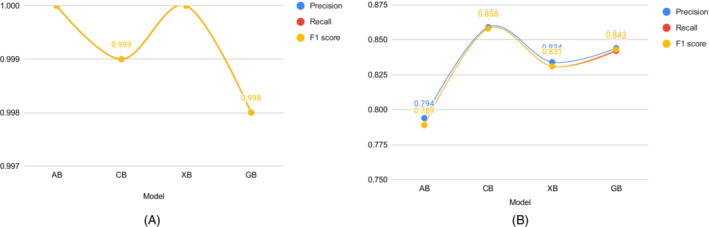
Performance of the algorithms: (A) Train data and (B) test data

The experiments are repeated 20 times for each run. The results will be different for each model. After that, the average scores for the evaluation metrics (precision, recall, and F1‐score) are considered. The average performance of the models on both the train as well as the test dataset is shown in Table 5 and Figure 10. It can be seen that CB and XB had almost the same train performance, whereas the AB model had comparatively lower train performance than other algorithms (see Figure 10A). It is evident from the test performance that, except for AB, all the algorithms were close considering performance. On the other hand, AB had lower test performance than other algorithms (see Figure 10B). For further evaluating the developed ML models, confusion matrices, as well as ROC curves,^50^ are also generated. The confusion matrices for the developed models on both the train as well as the test dataset for the AB, CB, GB, and XB are shown in Figures 2, 4, 6, and 8, respectively, while the ROC curves for the models are shown in Figures 3, 5, 7, and 9, respectively.

**TABLE 5:** Performance measures for the developed models

	Train			Test		
Model	Precision	Recall	F1‐score	Precision	Recall	F1‐score
AB	1.000	1.000	1.000	0.796	0.790	0.791
CB	0.999	0.999	0.999	0.858	0.859	0.859
XB	1.000	1.000	1.000	0.835	0.834	0.835
GB	0.993	0.992	0.992	0.845	0.843	0.842

### 4.1 Adaptive boosting

AB, which stands for adaptive boosting, is a meta‐learning ML algorithm used to boost the model's performance by fitting sequentially to the weak learning models such as decision trees.^51^ The output of the weak learners is combined into a weighted sum that represents the final output of the boosted classifier. This classifier is adaptive in nature because of the sharp pulling of weak learners where the instances are misclassified. Although it is sensitive to noisy data but less susceptible to the overfitting problem compared to other learning algorithms.^51^ On the training data, the precision, recall, and F1‐score for the AB model were 100% for all, whereas the precision, recall, and F1‐scores for the testing data were 79.4%, 78.9%, and 78.9%, respectively (see Table 5). The confusion matrices and the ROC curves for the AB model are shown in Figures 2 and 3, respectively. The micro and macro average of the AUC for the train ROC curve was 100%, while the micro and macro average of the ROC for the research ROC curve was 87% (see Figure 3).

### 4.2 CatBoost

CB is a widely used ML algorithm that efficiently handles categorical features and takes advantage of dealing with them during training instead of preprocessing time.^52^ It reduces the extensive need for hyperparameter tuning and has a lower chance of overfitting by applying ordered boosting to get the best result.^53^ The precision, recall, and F1‐score for the CB model on the training dataset were 99.9% for all, whereas the value of precision, recall, and F1‐score for the test dataset were 85.9%, 85.8%, and 85.8%, respectively (see Table 5). The confusion matrices and the ROC curves for the CB model are shown in Figures 4 and 5, respectively. It can be seen from the train ROC curve that the micro and macro average of the area under the curve (AUC) was 99%, whereas the micro and macro average ROC for the test ROC curve was 89% (see Figure 5).

### 4.3 Gradient boosting

GB is a widely used ML technique for solving regression and classification problems.^54^ This prediction algorithm is an ensemble form of weak prediction models or base learners, whereas the base learners are typically decision trees. Like other boosting methods, a stage‐wise fashioned model is used with an arbitrary differentiable loss function for optimizing the model. On the training dataset, the precision, recall, and F1‐score for the GB model were 99.8% for all, while the precision, recall, and F1‐score for the test dataset were 84.4%, 84.2%, and 84.3%, respectively (see Table 5). The confusion matrices and the ROC curves for the GB model are shown in Figures 6 and 7, respectively. The micro and macro average of the AUC for the train ROC curve was 95%, while the micro and macro average ROC for the test ROC curve was 72% (see Figure 7).

### 4.4 XGBoost

XB, which stands for extreme gradient boosting, is a kind of boosting algorithm that is recognized mostly for providing parallel tree boosting that is well used for solving data science problems accurately and efficiently in terms of speed as performance.^55^ This algorithm is also widely used to predict the online review polarity^56^ based on customer purchase decisions where the key features are extracted from the data based on ranking scores. The precision, recall, and F1‐score for the XB model on the training results were 100% for all, respectively, while the precision, recall, and F1‐scores for the testing data were 83.4%, 83.1%, and 83.1%, respectively (see Table 5). The confusion matrices and the ROC curves for the XB model are shown in Figures 8 and 9, respectively. The micro and macro average AUC for the train ROC curve was 100%, while the micro and macro average AUC for the test ROC curve was 87% (see Figure 9).

### 4.5 Comparative analysis of boosting algorithms

In our study, four different boosting algorithms are employed to classify tweet data. Applying each algorithm to the dataset led to different performances based on precision, recall, and F1‐score values. The highest F1‐score (85.8%) was achieved from the CB classifier from the test dataset. The F1‐score of GB, XB, and AB classifiers are 84.3%, 83.1%, and 78.9%, respectively. Similarly, recall values of the applied algorithms followed the same performance pattern as the F1‐score (see Table 5). Again, the CB classifier achieved the highest precision score (85.9%) among the other classifiers, whereas GB, XB, and AB achieved a precision score of 84.4%, 83.4%, and (79.4%) on the test dataset, respectively. On the other hand, the highest F1‐score (100%) was achieved by the AB and XB classifier other than the CB classifier (99.9%) on the training dataset while the GB classifier achieved the lowest F1‐score of 99.8%. Similarly, recall and precision values have followed the same performance as the F1‐score for the applied algorithms on the training dataset (see Table 5). Figure 10 depicts all three evaluation metrics of precision, recall, and F1‐score.

## 5 KEY INSIGHTS FROM UK, USA, AND BANGLADESH TWEETS

**FIGURE 11:**
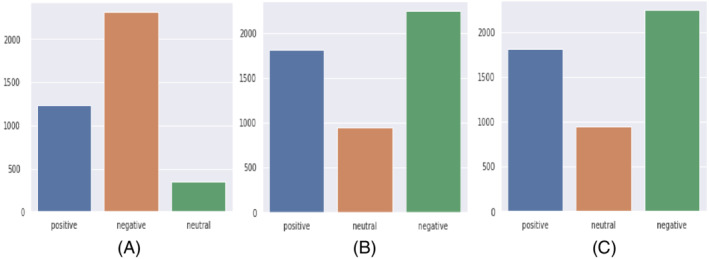
Frequency of the classified tweets: (A) Bangladesh, (B) UK, and (C) USA

To explore how the public sentiment changed for three different countries with respect to time during the COVID‐19 pandemic, the collected tweets were classified into three categories (positive, negative, and neutral) using different ML models. Figure 11 presents a bar chart where different sentiments are plotted against the number of frequencies in the predicted tweets. It is evident from the graph that public sentiment was mostly negative for all the countries, followed by positive and neutral sentiment. The python pandas^¶^ and matplotlib^#^ implementation packages of the python programming language were used for data analysis and visualization to explore the sentiment of people during the COVID‐19 pandemic as presented in the following subsections.

### 5.1 Frequently stated words

**FIGURE 12:**
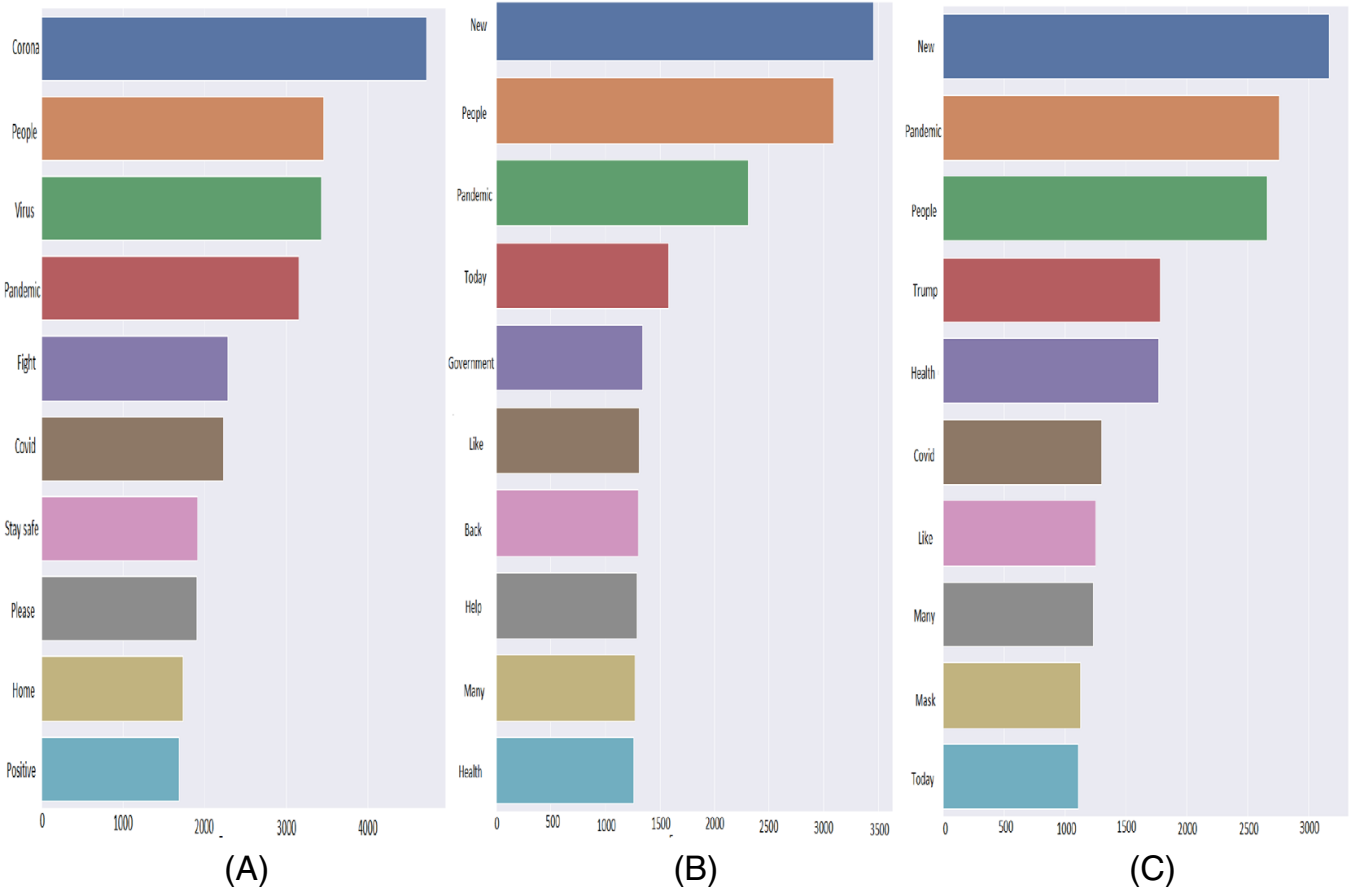
Ten most frequent words: (A) Bangladesh, (B) UK, and (C) USA

The most frequent 10 words from all the tweets and their number of occurrences in the dataset are plotted for Bangladesh, the UK, and the USA in Figure 12. Mostly observed words from Bangladeshi tweets were “corona,” “people,” “virus,” “pandemic,” and “fight'. The presence of these words in BD tweets indicates that on the one hand, people were concerned about the COVID‐19 pandemic, on the other hand, people were optimistic about fighting against the pandemic. The most observed words from UK tweets were “new,” “people,” “pandemic,” “today,” and “government,” where the presence of these words indicates that people were concerned about the new normal life during the ongoing COVID‐19 pandemic, also it can be seen that people talked about the steps taken by the government to curb the spread of the virus. Again, the following words were observed from USA tweets: “new,” “pandemic,” “people,” “trump,” and “health'. Thus, these words indicate that the people of the USA stated their concern about the health issues of the citizens in their new normal life during the pandemic. Interestingly the name of the former US president “trump” was enlisted in one of the most frequent words. By analyzing the most frequent words from three countries, common words were found such as: “covid,” “pandemic,” and “corona'. As tweets are collected in the COVID‐19 context, thus it can be easily understood that these words will be most frequent for all the countries.

### 5.2 Word clouds from different sentiments

**FIGURE 13:**
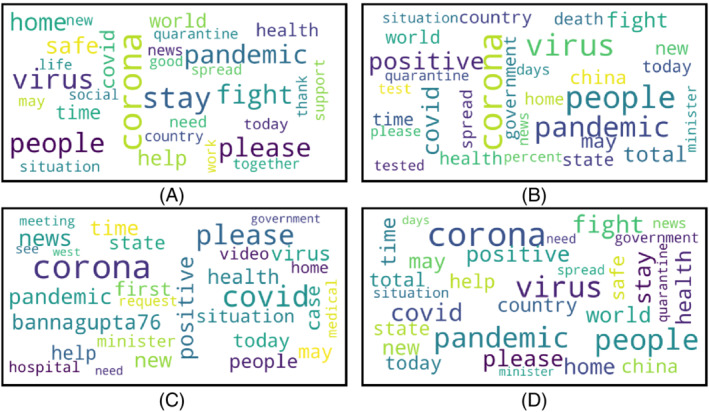
Word clouds generated from BD tweets: (A) Word cloud for positive tweets, (B) word cloud for negative tweets, (C) word cloud for neutral tweets, and (D) word cloud for all tweets

**FIGURE 14:**
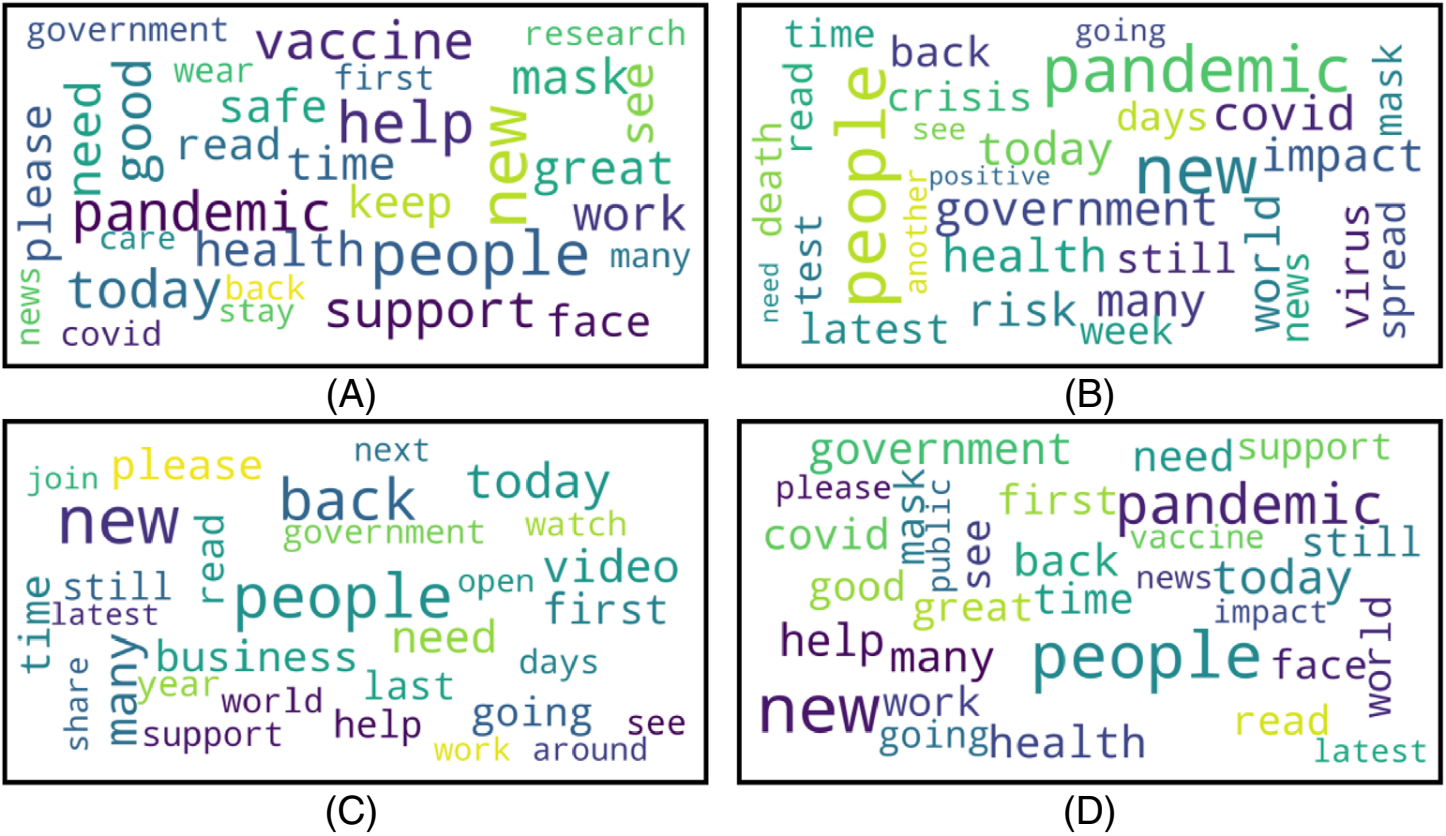
Word clouds generated from UK tweets: (A) Word cloud for positive tweets, (B) word cloud for negative tweets, (C) word cloud for neutral tweets, and (D) word cloud for all tweets

**FIGURE 15:**
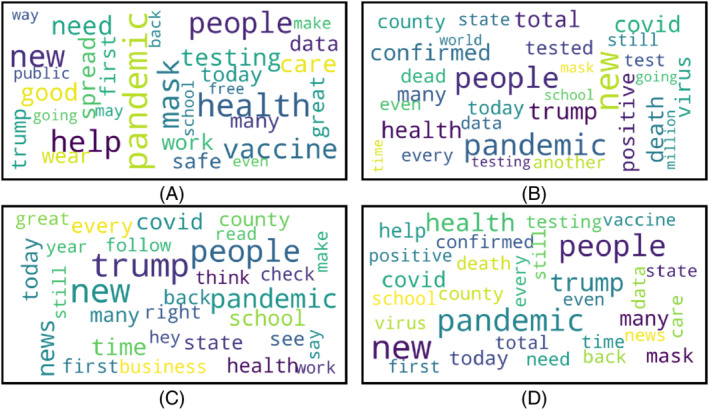
Word clouds generated from USA tweets: (A) Word cloud for positive tweets, (B) word cloud for negative tweets, (C) word cloud for neutral tweets, and (D) word cloud for all tweets

Word clouds are generated for positive, negative, neutral, and the mixture of all sentiments for three different countries. The word clouds for Bangladesh, the UK, and the USA are presented in Figures 13, 14, 15, respectively.

#### 5.2.1 Bangladesh

Considering positive, negative, and neutral sentiment from the annotated tweets data for Bangladesh three separate word clouds are presented in Figure 13A–C, respectively. Some positive words from Bangladeshi people were “corona,” “virus,” “pandemic,” “support,” and “quarantine'; which means Bangladeshi people not only worried (negative) about the COVID‐19 pandemic but also showed hope‐full and expressed positive sides of the pandemic. People were positive regarding being safe and supportive of each other. The presence of the word “quarantine” in positive sentiment indicates that people were positive about quarantining themselves to fight against the pandemic.

Negative word cloud mostly contains the worlds: “pandemic,” “corona,” “tested,” “positive,” “people,” and “virus'. However, it can be seen that the word “corona” has the same number of occurrences in the tweets of positive, negative, and neutral sentiments. During the COVID‐19 pandemic, the social media platforms were flooded with the news of more and more people being tested COVID‐19 positive, and the presence of the words like “tested positive,” “hospital,” and “health” in the word cloud of negative tweets means positive sense in literal meaning, but in the practical sense, these words were used negatively in the tweets. This news hampered the mental health of mass people.^57^, ^58^ People expressed “neutral” sentiment regarding “covid,” “cases,” “government,” “minister,” and “hospital'. The government had to take a lot of steps to control COVID‐19,^59^ and it can be observed that the overall sentiment of people regarding the government was neutral. Lastly, some words with mixed sentiments were “stay,” “home,” “safe,” and fight,” which indicates that the most discussed topics of Bangladeshi people were about staying safe in‐home and fighting against the pandemic.

#### 5.2.2 UK

The word clouds for the tweets of the positive, negative, and neutral sentiments are shown in Figure 14 for the UK. The positive words from the UK were “vaccine,” “support,” “government,” “mask,” “wear,” “stay,” and “safe' (see Figure 14A). The presence of the word “vaccine” in a positive word cloud means that people of the UK took “vaccine” positively. Other positive words mean people were positive about wearing masks and supportive of each other for fighting against COVID‐19. Few observed negative words were “pandemic,” “crisis,” “risk,” “virus,” and “spread” (see Figure 14B). It can be observed that the word “government” is larger in the negative word cloud than the positive word cloud, which means that the sentiment of the UK people was mostly negative toward their government than the positive sentiment. People also expressed negative concerns thinking about the risk to their lives as well as the economic^60^ crisis created due to the pandemic. The words representing neutral sentiment were “help,” “support,” “share,” “government,” “watch,” and “video'. The word “government” is also present in the neutral sentiment. While the word size is smaller than the word in positive and negative sentiment, it can be said that some people also expressed neutral sentiment toward the government.

#### 5.2.3 USA

Word clouds for positive, negative, neutral, and all sentiments are presented in Figure 15, respectively. Some words in the positive sentiment were “pandemic,” “vaccine,” “help,” “need,” and “trump' (see Figure 15A). The presence of the word “trump” in the tweets of positive sentiment refers to the fact that people's sentiment was positive regarding former US President Donald Trump. Similarly, the word “vaccine” means that people's sentiments were mostly positive regarding vaccination. People also expressed a positive attitude toward helping each other in the pandemic. Interestingly, the word “pandemic” can be observed in positive sentiment, which means people also expressed the positive side of the pandemic like less environmental pollution, less traffic on the roads, and so forth. However, the word “pandemic” can also be observed in negative and neutral word clouds, although the size of these words in these word clouds was less small than the word in the word cloud of positive sentiment. Some other words like “corona,” “tested,” “positive,” “minister,” and “quarantine” were observed for negative sentiment tweets. It can be seen that although people expressed a negative attitude on more and more the increasing COVID‐19 cases, people were not supportive of the government's steps and did not take the idea of quarantining themselves positively. Nonetheless, people expressed neutral sentiment toward “government,” “hospital,” “meeting,” and “medical'. The pandemic created a huge crisis in the supply of medical equipment and hospital facilities, but most people expressed neutral thoughts on these issues.

### 5.3 Sentiment over time

**FIGURE 16:**
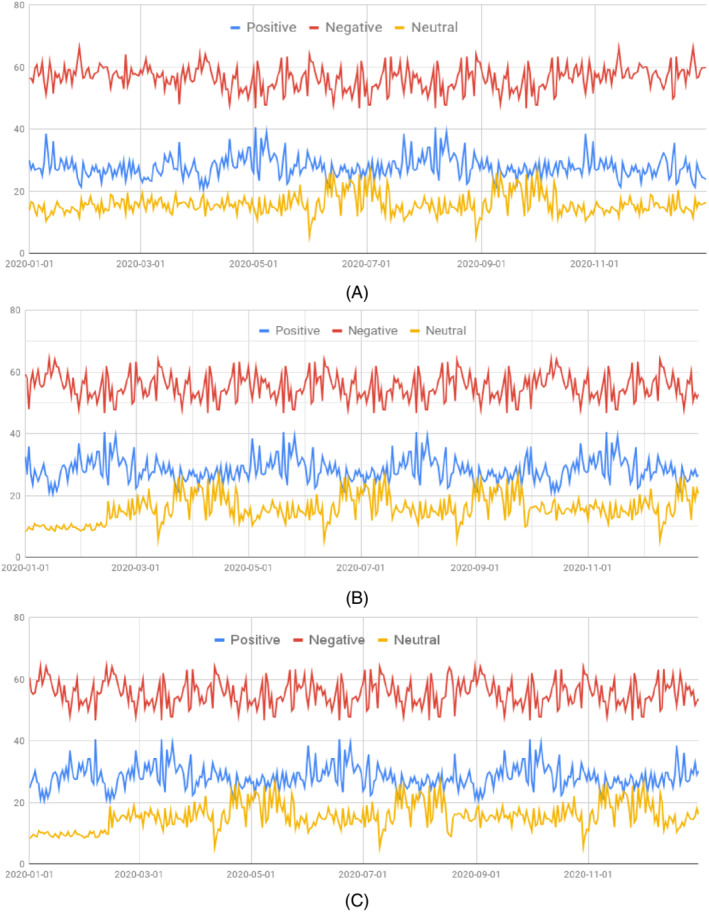
Public sentiment over time: (A) BD, (B) UK, and (C) USA

The public sentiment during a pandemic varies over time for a few reasons, such as infection rates, availability of health services and medical equipment, unemployment, domestic violence during the pandemic, development of vaccines, and other countermeasures taken by the governments. During the COVID‐19 pandemic, public sentiment was found to change over time on the percentage of positive, negative, and neutral sentiment (see Figure 16), and the percentage of each of the sentiments is plotted with respect to time for BD, UK, and the USA in Figure 16A–C, respectively, in. By observing the public sentiment overtime for the Bangladeshi sentiment in Figure 16A, it can be said that percentage of positive, negative, and neutral sentiment was initially very high, whereas the percentage of negative sentiment was higher than neutral, and the percentage of neutral sentiment was higher than positive sentiment. Although a lot of ups and downs can be observed in the percentage of the positive, negative, and neutral sentiment of people, the overall sentiment of people became almost stable after a fixed amount of time.

Considering the public sentiment over time for the UK in Figure 16B, it can be seen that three said sentiment of people raises initially as time passes on. After reaching the peak point amount, the negativity among people decreases. However, another rise in the percentage of negative sentiment can be observed after the middle of the year. For the UK sentiment, the number of ups and downs of public sentiment was comparatively lesser than BD sentiment.

For the sentiment timeline of the USA, which is shown in Figure 16C, a comparatively fewer number of ups and downs can be observed compared to the other two countries. Here also, the negativity among people was initially very high, but unlike the other two countries, the negativity soon decreased and the relation of the positive‐negative and neutral sentiment almost maintained a stable relationship for a long period of time. At the end of the year, it is observed that the percentage of negativity among people started increasing drastically.

### 5.4 Sentiment against important concern

**FIGURE 17:**
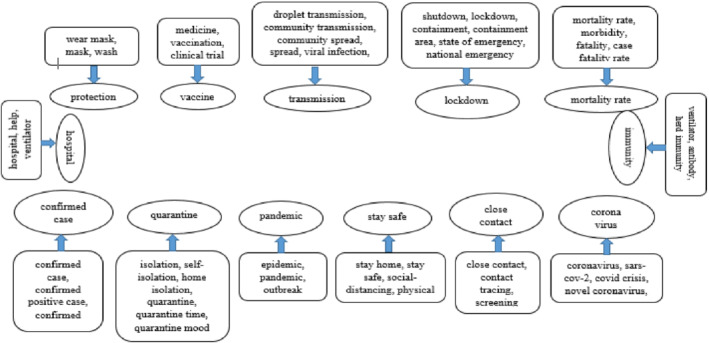
Clustering of concern topics based on similar meaning in COVID‐19

Some important concerns have been identified regarding COVID‐19 from all the word clouds as shown in Figures 13, 14, 15. The COVID‐19 pandemic has created a flood of new words and phrases in both English and other languages. The concerned topics are selected based on these frequently used new relevant words that have a huge impact to express sentiment in the tweets. The concern topics are “vaccine,” “medicine,” “hospital,” “government,” “social distancing,” “lockdown,” “shutdown,” “stay home,” “stay safe,” “wear mask,” “quarantine,” “epidemic,” “pandemic,” “outbreak,” “containment,” “hospital,” “help,” “droplet transmission,” “viral infection,” “incubation,” “confirmed case,” “confirmed positive case,” “close contact,” “contact tracing,” “screening,” “coronavirus,” “covid crisis,” “isolation,” “self isolation,” “home isolation,” “social distancing,” “physical distancing,” “ventilator,” “antibody,” “herd immunity, “mortality rate,” “morbidity,” “fatality,” “case fatality rate,” “state of emergency,” “national emergency,” “clinical trial,” “vaccination,” and so forth. We have further investigated the showed keywords for clustering based on their thematic similarity which is shown in Figure 17. These findings showed that sentiment against important (clustered) concerns varied from country to country. This variation depicted the people's acceptance, thoughts, and emotions toward the COVID‐19 pandemic. The sentiment against important concerns for each country is discussed in the following subsections.

#### 5.4.1 Bangladesh

**FIGURE 18:**
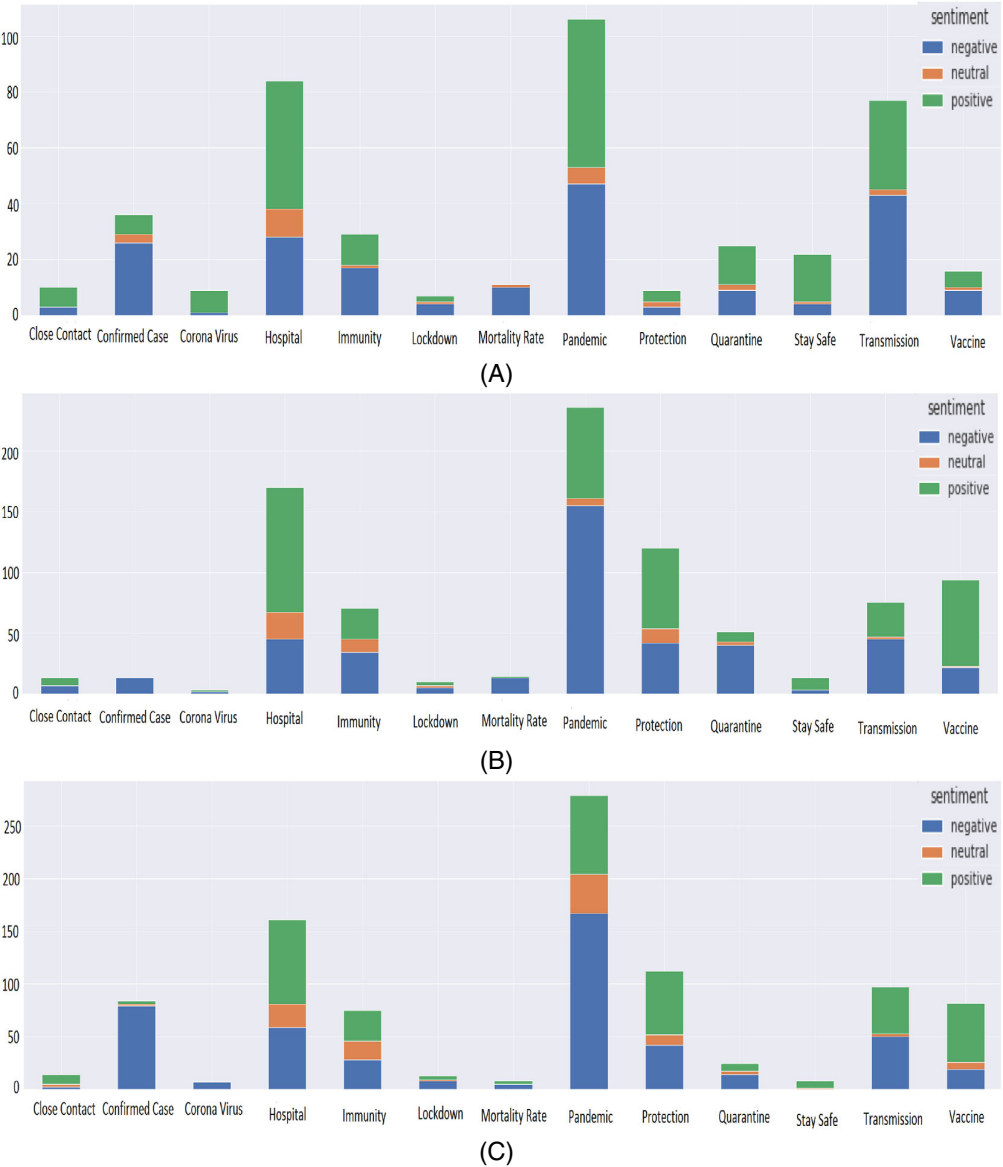
Sentiment against important concerns for: (A) BD, (B) UK, and (C) USA

The important concerns revealed in the context of Bangladesh are depicted in a vertically stacked bar graph in Figure 18A. The figure showed that the word “pandemic” is used more as a negative sentiment than positive sentiment in Bangladesh compared to the USA and UK (see Figure 18B,C). Similarly, the word “quarantine” is mostly used more positive than negative sentiment in Bangladesh while in the other two countries, the word “quarantine” showed much a negative sentiment. The possible reasons for considering “pandemic” positively could be the following: At the initial stage of the COVID‐19 crisis, specifically, the young people including university and school students have taken the pandemic positively as the exams and events were closed on March 16, 2020 in Bangladesh but later on it is found that students of graduate level of education experienced high levels of stress.^61^ Again, the countrywide lockdown has made significant improvements in the air quality of the capital city Dhaka as well as reduced different environmental pollution.^62^ The word “vaccine” showed negative sentiment since most of the people of Bangladesh are getting over their initial fear in COVID‐19 vaccination. In case of words like “confirmed case,” “mortality rate,” “coronavirus,” “lockdown,” and “transmission” showed negative sentiment with respect to other sentiment labels like positive and neutral. The people of the UK and USA showed the same negative sentiment as Bangladesh for those words. However, the word “hospital” shows opposite trends to the word “pandemic'; where positive sentiment is more dominant with respect to negative and neutral sentiment in Bangladesh. The words “immunity,” “stay safe,” “protection,” and “mask” follow the same trend as the word “hospital'.

#### 5.4.2 UK

The important concerns for the UK are presented in Figure 18B. The term “pandemic” has more negative sentiment than the other sentiment labels in the UK. Unlike the UK, the USA followed the same trends for this word, whereas Bangladesh showed less negativity. On the other hand, the word “quarantine” is mostly used negatively in the UK and the USA, whereas Bangladesh showed a more positive sentiment regarding this concern. The words “hospital,” “immunity,” “protection,” “stay safe,” “mask,” and “vaccine” have shown positive sentiment compared to other sentiment labels in the UK. On the contrary, negative sentiment is more prevalent over the words like “confirmed case,” “mortality rate,” “coronavirus,” “lockdown,” and “transmission” in the UK. The analysis of the important concern topics showed that the people of the UK were more concerned compared to other countries.

#### 5.4.3 USA

The important concerns for the USA are presented in Figure 18C. The word “pandemic” has shown more negative sentiment in the USA compared to other sentiment labels. Similar trends were noticed in the UK (see Figure 18B) for “pandemic” while Bangladesh showed less negativity. Similarly, the word “quarantine” is more negatively used than positive sentiment, whereas Bangladesh showed much more positive sentiment compared to negative sentiment. The words “hospital,” “immunity,” “stay safe,” “protection,” “mask,” and “vaccine” have shown positive sentiment compared to other sentiment labels. On the other hand, negative sentiment is more dominant over the words like “confirmed case,” “mortality rate,” “coronavirus,” “lockdown,” and “transmission” in the USA. These findings indicated that the people of the USA had taken the COVID‐19 situation more seriously than a country like Bangladesh.

## 6 DISCUSSION

### 6.1 Results

This article focused on sentiment analysis of COVID‐19‐related tweet data by applying several ML models. This research yielded the following outcomes. First, among several ensemble‐based classifier models, CatBoost (F1‐score 85.8%) was the best‐performed ML model to classify tweets into positive, negative, and neutral sentiment. The CatBoost model can be regarded as the baseline learning method for sentiment analysis. Second, sentiment variation of the people of three different countries from three separate geographical locations is analyzed, and it was identified that people's sentiment in those countries regarding the pandemic was mostly negative, followed by positive and neutral. Third, this study identified some important concerns, such as lockdown, quarantine, hospital, mask, and vaccine, that impact mostly the sentiments across three countries due to the COVID‐19 pandemic.

### 6.2 Key contributions

The contributions of this research, in contrast to prior studies, can be summarized as follows. First, the majority of the research looked at sentiment from COVID‐19 tweets in the context of a single nation,^7^, ^10^ while this research analyzes public sentiment from a cross‐country perspective. Second, various research used various ML approaches to analyze public perceptions, while this research used multiple ML approaches to determine which one worked best, and the best performing methodology was used to analyze tweets. Third, the majority of studies that assess public opinion were published in the early or middle stage of the COVID‐19 pandemic. Thus, these studies fail to capture the whole timeline of the COVID‐19 pandemic, while this study captured the public sentiment considering the whole year of 2020 (January 1st to December 31).

### 6.3 Limitations and future work

There are a few limitations to this research. First, the analysis may represent public sentiment based on a biased sample of the tweets obtained from three geographical locations. Second, in this research, a random 80–20 train–test split is applied to the dataset. As the dataset is split randomly, it would be better if k‐fold cross‐validation was chosen to perform the experiments. Third, only USE is used to do the text embedding. Therefore, future studies may focus on applying k‐fold cross‐validation techniques while trying different encoders, such as Bert, Word2vec, for text embedding to get relevant results. Explainable artificial intelligence approaches will also need to be investigated.^63^

### 6.4 Conclusion

During critical periods like the COVID‐19 pandemic, the public mood directly influences officials' policies. Policymakers do any authoritative decision or action will be in vain without the cooperation of the people because the people hold the most power in any country on the planet. Thus, analyzing public perception is emergent in times of pandemics. This research analyzed the worldwide public perceptions and the reasons behind the variations of public sentiment, which will greatly contribute to the government, policymakers, and health workers to take any decisions and increasing overall public awareness about COVID‐19 and future pandemics.

## AUTHOR CONTRIBUTIONS

Muhammad Nazrul Islam conceived the study design and oversaw its writing. The initial draft of the article was prepared by Md Mahbubar Rahman and Nafiz Imtiaz Khan. The data collection was done by Md Mahbubar Rahman. Nafiz Imtiaz Khan was in charge of the coding. Mohiuddin Ahmed, Muhammad Nazrul Islam, and Iqbal H. Sarker were involved in the data analysis. Muhammad Nazrul Islam, Md Mahbubar Rahman, Nafiz Imtiaz Khan, and Mohiuddin Ahmed took part in revising and drafting the final article. All authors read and approved the final article.

## CONFLICT OF INTEREST

The authors declare no conflict of interest.

### PEER REVIEW

The peer review history for this article is available at https://publons.com/publon/10.1002/eng2.12572.

## Data Availability

The authors elect not to share data.

## ENDNOTES

*
https://www.who.int/

†
https://www.tensorflow.org/tutorials/text/word2vec

‡
https://paperswithcode.com/method/bert

§
https://www.tensorflow.org/hub/tutorials/semantic_similarity_with_tf_hub_universal_encoder

¶
https://pandas.pydata.org/

#
https://matplotlib.org/
